# Camera‐Trap Evidence of *Myricaria* sp. Consumption and Head‐Rubbing by a Wild Snow Leopard (*Panthera uncia*) in an Alpine Ecosystem

**DOI:** 10.1002/ece3.73765

**Published:** 2026-06-01

**Authors:** Kodzue Kinoshita, Hiroto Yoshimura, Kubanychbek Zhumabai uulu, Koustubh Sharma, Aibek Sergek uulu, Emil Djaparov, Kurmanbek Tolok uulu, Dale M. Kikuchi

**Affiliations:** ^1^ Graduate School of Asian and African Area Studies Kyoto University Kyoto Japan; ^2^ Wildlife Research Center Kyoto University Kyoto Japan; ^3^ Snow Leopard Foundation in Kyrgyzstan Bishkek Kyrgyzstan; ^4^ Snow Leopard Trust Seattle Washington USA; ^5^ Department of Protected Areas and Biodiversity Conservation The Ministry of Natural Resources, Ecology, and Technical Supervision Naryn Kyrgyzstan; ^6^ No Institutional Affiliation Kaiyrma Kyrgyz Republic; ^7^ Department of Earth System Science, Faculty of Science Fukuoka University Fukuoka Japan

**Keywords:** camera‐trap, eating plant, feeding behavior, felids, *Myricaria*, obligate carnivore

## Abstract

Plant consumption by felids is widely reported; however, direct behavioral evidence in wild individuals remains scarce. A camera‐trap was used to document plant‐directed behaviors by wild snow leopards ( *Panthera uncia* ) toward *Myricaria* sp. and to compare responses of sympatric mammals. A camera was deployed at approximately 2580 m in the Shamshy co‐managed nature protected area, Kyrgyzstan, recording 60‐s videos upon motion detection across 412 monitoring days. In total, 1065 videos were obtained, including 131 mammal encounters representing nine wild species and four domestic species. Plant consumption was observed in snow leopards, domestic horses ( *Equus ferus caballus* ), Siberian ibex ( *Capra sibirica* ), and domestic cattle (*Bos taurus*); however, *Myricaria* sp. ingestion occurred only in snow leopards and horses. A single adult male snow leopard was recorded on 15 occasions across three non‐consecutive days. Across 15 videos, feeding on *Myricaria* sp. occurred during 14, typically following sniffing (11/15) and then, often, head‐rubbing (7/15). The snow leopard progressively consumed branches from distal tips toward the proximal base. These events occurred in winter and early spring when *Myricaria* sp. bore mostly stubbles and stems. Horses appeared on six non‐consecutive days (50 videos) and fed on *Myricaria* sp. only during the leafy period recorded on 1 day (14 videos) on 25th September 2022; seven of 18 passing horses fed, defoliating shrubs. Domestic dogs and ibexes sniffed *Myricaria* sp. in three and four videos, respectively, but did not consume it. These observations provide the first camera‐trap evidence of *Myricaria* sp. consumption by wild snow leopards and reveal associated sniffing followed by feeding and then head‐rubbing, suggestive of olfactory assessment and also potential marking. Species‐specific use of *Myricaria* sp. indicates roles beyond forage. The findings motivate targeted chemical and physiological studies to clarify functional drivers (e.g., gastrointestinal, self‐medication, oral stimulation, or scent‐marking) of plant‐directed behaviors in felids.

## Graphical abstract

Camera‐trap footage from the Shamshy co‐managed nature protected area, Kyrgyzstan, provides the first direct evidence of a wild snow leopard ingesting woody *Myricaria* sp., often alongside sniffing and head‐rubbing. Species‐specific use—snow leopards feeding in leaf‐poor periods vs. horses only in leafy periods—suggests functions beyond forage and motivates chemical/physiological tests.

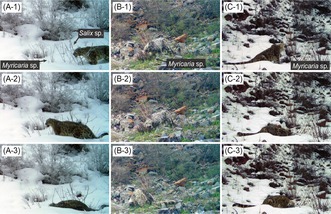

## 1 Introduction

**FIGURE 1:**
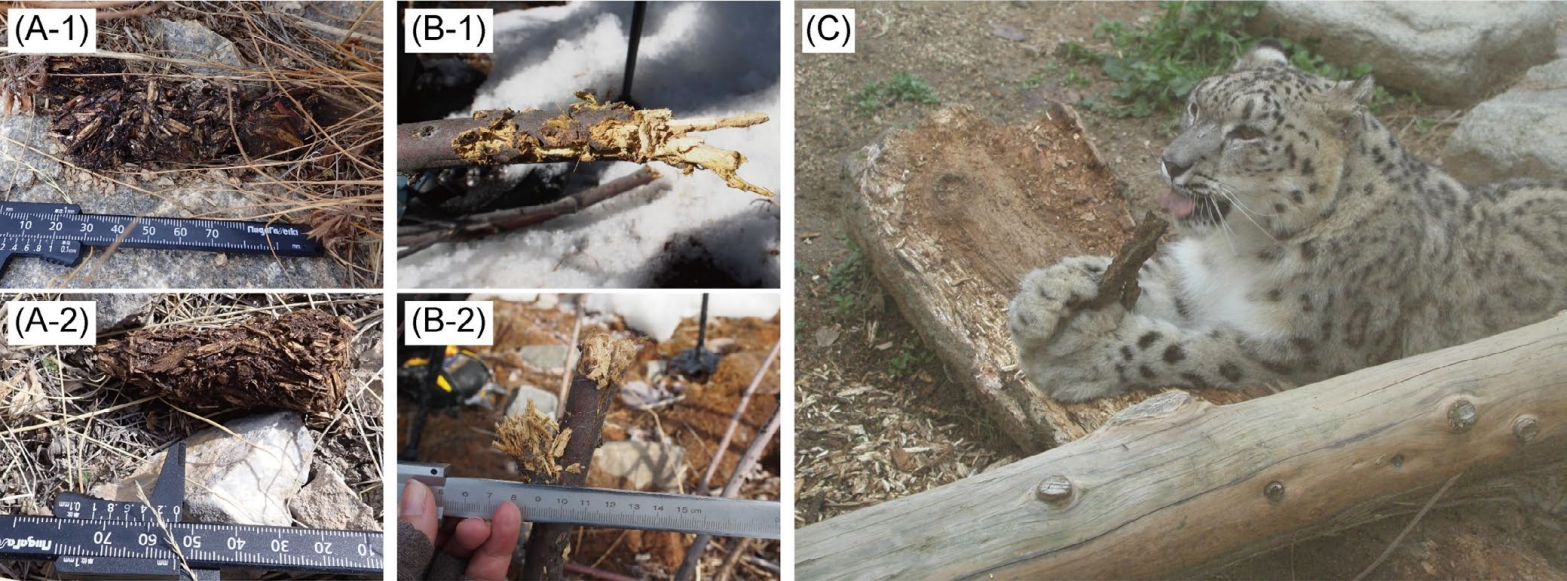
Images supporting the hypothesis of plant consumption by snow leopards: (A‐1, 2) Fecal samples from snow leopards collected in the Shamshy co‐managed nature protected area, Kyrgyzstan in November 2022. Samples were identified through DNA analysis and numerous small branches consistent with *Myricaria* sp.; (B‐1, 2) The *Myricaria* sp. plant exhibiting feeding marks at the same study site, taken in March 2024; (C) A captive female snow leopard gnawing on wood fragments at Kobe Oji Zoo in December 2006.

**FIGURE 2:**
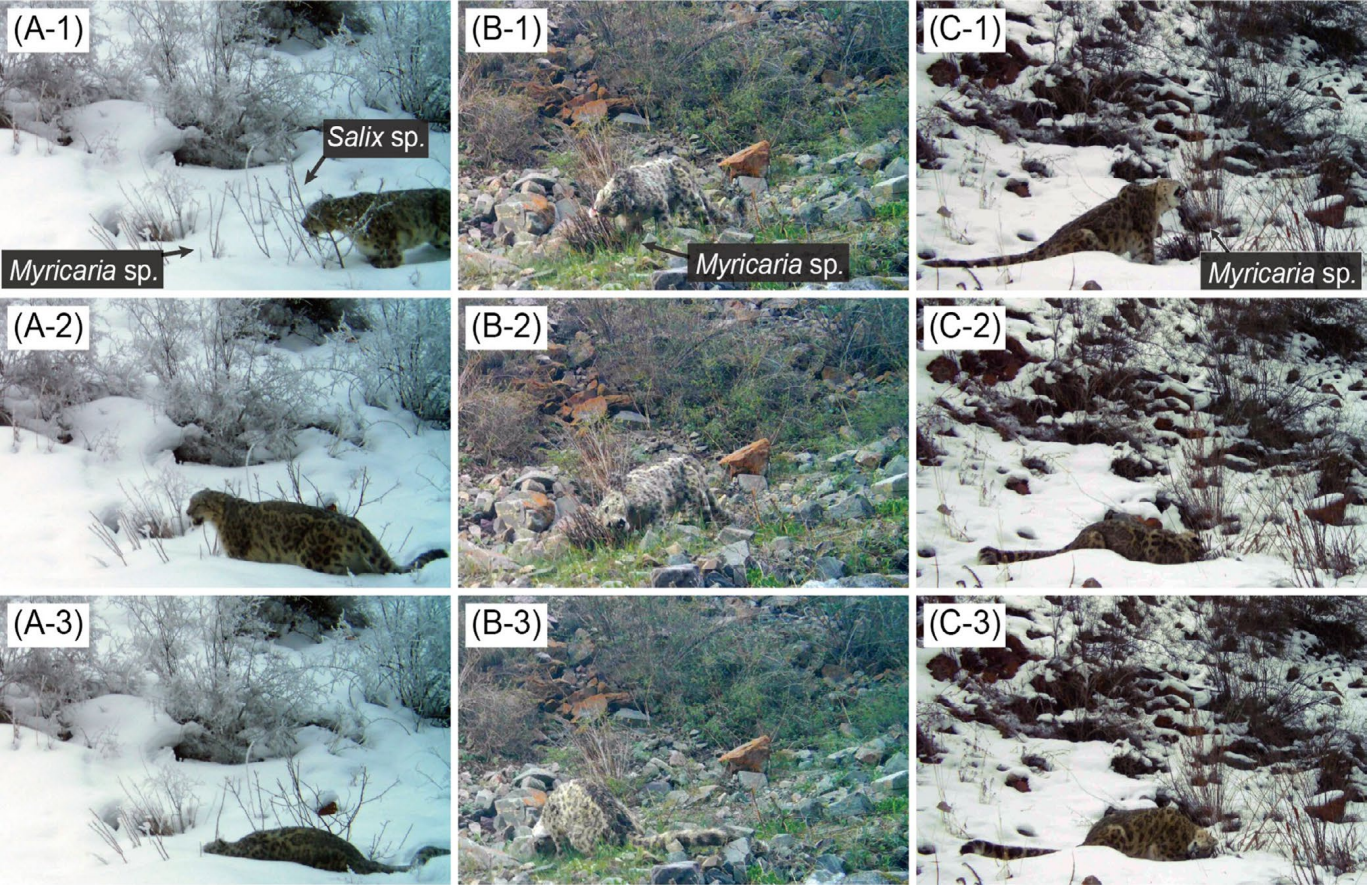
Sequential images of a male snow leopard in three separate occurrences, taken in the Shamshy co‐managed nature protected area, Kyrgyzstan: (A) 9th December 2022: (A‐1) sniffing *Salix* sp., (A‐2) biting *Myricaria* sp., and (A‐3) gnawing and consuming the branch down to its base; (B) 24th May 2023: (B‐1) biting *Myricaria* sp., (B‐2) gnawing and consuming toward its base, and (B‐3) engaging in head‐rubbing against the plant; (C) 25th November 2024: (C‐1) biting *Myricaria* sp., (C‐2) gnawing and consuming toward its base, and (C‐3) engaging in head‐rubbing against the plant.

While plant consumption by felids is widely reported despite their obligate carnivorous nature (Van Valkenburgh 1989; Legrand‐Defretin 1994; Wortinger 2006; Hart et al. 2021; Yoshimura et al. 2021; Bensel et al. 2025), direct behavioral evidence in wild individuals remains scarce. Snow leopards ( *Panthera uncia* ), whose habitats are usually sparsely vegetated alpine regions, have been documented to consume grass via fecal analysis (Oli et al. 1993; Anwar et al. 2011; Shehzad et al. 2012; Wegge et al. 2012; Devkota et al. 2013; Jumabay‐Uulu et al. 2014; Yoshimura et al. 2024). In particular, fragments of *Myricaria* species are often detected in snow leopard feces from the Tien Shan and Himalayan regions (Lovari et al. 2013; Jumabay‐Uulu et al. 2014; Yoshimura et al. 2024). This genus, belonging to the *Tamaricaceae* family, is distributed across many regions within the range of the snow leopard (Zhang et al. 2014). Plant matter including leaves and branches in their feces (Figure 1A‐1,2) and branches exhibiting bite marks characteristic of a carnivorous species (Figure 1B‐1,2) are frequently found, and we have previously observed a captive female snow leopard gnawing on wood fragments (Figure 1C). However, direct behavioral evidence in wild snow leopards has never been documented. Although such consumption is believed to be deliberate for carnivores, including snow leopards (Franck and Farid 2020), accidental ingestion during feeding on prey cannot be fully ruled out. Here, to better understand the nature of these interactions, we analyzed the behaviors of wild snow leopards toward *Myricaria* sp., including feeding, sniffing, and head‐rubbing. We also examined how herbivorous species responded to *Myricaria* sp., focusing on differences in feeding behavior, seasonal use, and olfactory investigation. This study provides the first direct camera‐trap evidence of plant‐directed behaviors in wild snow leopards and offers comparative insights into how both carnivores and herbivores engage with the same plant species in a shared habitat.

## 2 Methods

This study was conducted in Kyrgyzstan's Shamshy co‐managed nature protected area, where hunting is prohibited, and resource use, including livestock grazing, is regulated. Shamshy is characterized by a heterogeneous mountainous landscape transitioning from riparian meadows and forests to grasslands and steep, rocky high‐elevation peaks with glacial features. A Browning BTC‐7 4K camera‐trap (Browning, Morgan, USA) was opportunistically deployed at approximately 2580 m along a potential snow leopard trail, where *Myricaria* sp. occurred along the river corridor and was surveyed over a 5 m stretch of the left bank upstream from 2457 m, yielding 41 individuals (*cf. bracteata* or *germanica*, based on bract morphology and inflorescence structure). Among these, the mean height of these individuals was 161.4 ± 51.8 cm, with a mean canopy spread of 218.5 ± 11.5 cm (major axis) and 164.2 ± 92.2 cm (minor axis) at snow leopard shoulder height (about 60 cm). Five individuals showed feeding signs likely attributable to carnivores, with the affected branches measuring 1.0 ± 0.4 cm in diameter. The camera was installed at the site with the highest elevation among these locations. The camera operated during the following periods: from 8th September 2022 to 9th December 2022; from 4th May 2023 to 22nd September 2023; and from 15th September 2024 to 10th March 2025. It was set to record 60‐s videos upon motion detection. Observed species were identified, and the one–zero sampling method was applied to record their behaviors by assigning a “one” for the occurrence or a “zero” for the non‐occurrence of selected behaviors during each 60‐s video recording. To ensure the independence of observations, an “Independent encounter” was defined based on a daily threshold; multiple video clips of the same species recorded within the same calendar day were treated as a single independent encounter. To categorize these behaviors objectively, we developed a standardized ethogram (Table S1) based on Kinoshita et al. (2009). For the present analysis, we focused on three key plant‐related behaviors: “Eating”, “Sniffing”, and “Rubbing”. Following these established criteria, “Sniffing” was strictly defined as the individual positioning its nose within approximately 10 cm of an object and remaining stationary for at least 3 s. “Eating” was recorded only when the sequence of mastication and subsequent ingestion (swallowing) was clearly observed. “Rubbing” was defined as the deliberate movement of the head or neck against the plant surface. These specific behaviors were selected to investigate the nature of the snow leopard's interaction with *Myricaria* sp.

## 3 Results

Over the entire monitoring period of 412 days, 1065 videos were captured, including 167 of birds and 131 of mammal encounters. The camera recorded nine species of wild mammals and four species of domestic animals (Table 1). Among these, domestic cattle ( *Bos taurus* ; hereafter “cattle”), domestic horses ( *Equus ferus caballus* ; hereafter “horses”), Siberian ibexes ( *Capra sibirica* ; hereafter “ibex”), and snow leopards consumed plants, whereas *Myricaria* sp. was eaten exclusively by horses and snow leopards (Table 1).

**TABLE 1:** Summary of video recordings showing various mammal species consuming *Myricaria* sp. and other plant types. Multiple clips of the same species recorded within a single calendar day were treated as a single independent encounter.

Mammal	Independent encounters	Videos	Videos	Videos	Videos	Videos
			Eating *Myricaria* sp.	Eating plants other than *Myricaria* sp.	Sniffing *Myricaria* sp.	Rubbing *Myricaria* sp.
Elk	1	1	0	0	0	0
Siberian ibex	**11**	**25**	**0**	**3**	**4**	**0**
Wild boar	1	2	0	0	0	0
Eurasian red squirrel	12	14	0	0	0	0
Mountain weasel	2	2	0	0	0	0
Stone marten	2	2	0	0	0	0
Red fox	5	5	0	0	0	0
Golden jackal	1	1	0	0	0	0
Snow leopard	**3**	**15**	**14**	**0**	**11**	**7**
Domestic cattle	**1**	**3**	**0**	**2**	**0**	**0**
Domestic sheep	1	1	0	0	0	0
Domestic horse	**6**	**50**	**14**	**15**	**0**	**0**
(with Human)	(5)	(9)	(0)	(0)	(0)	(0)
Domestic dog	**5**	**7**	**0**	**0**	**3**	**0**
(with Human)	(5)	(7)	(0)	(0)	(3)	(0)

**FIGURE 3:**
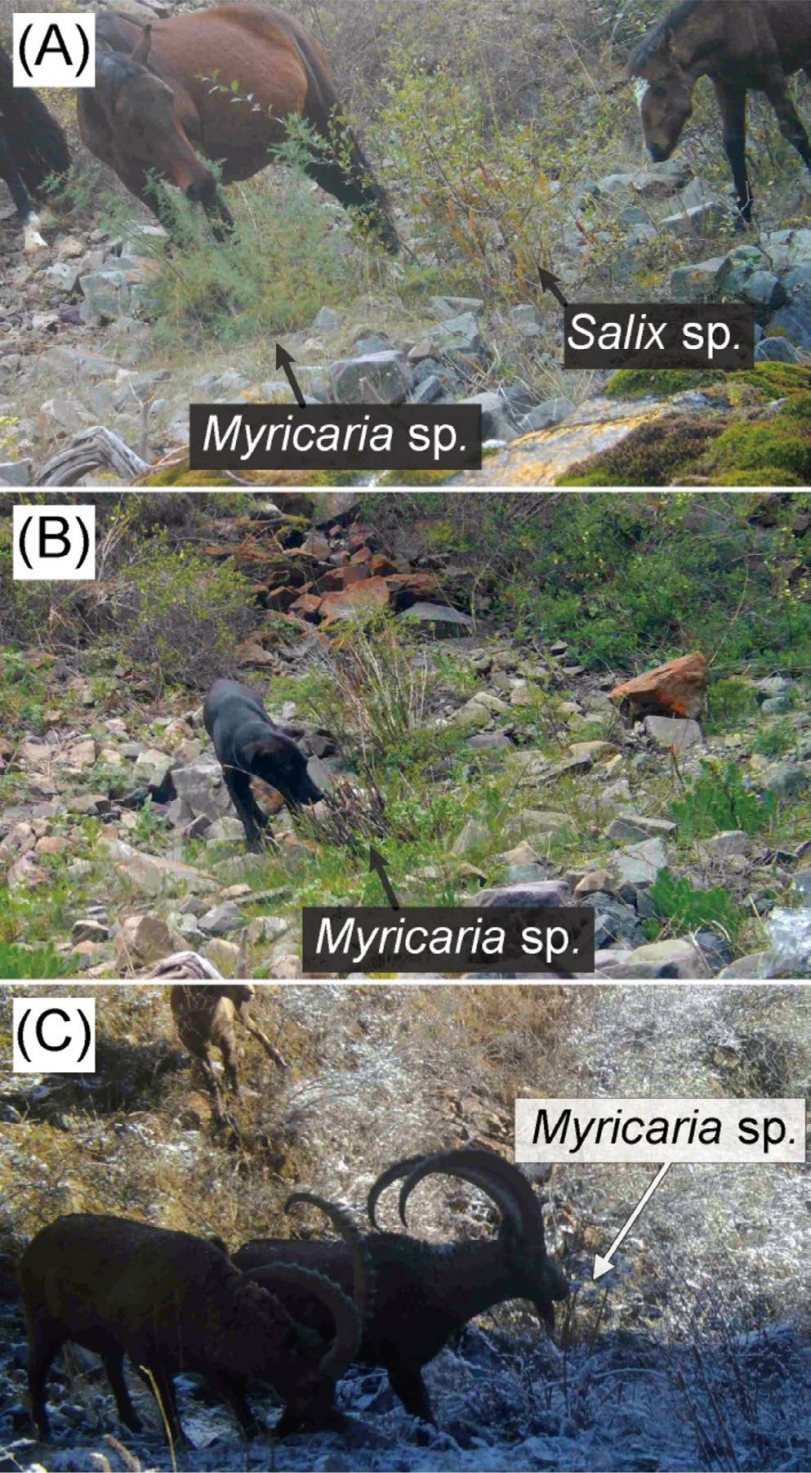
Camera‐trap images of species other than snow leopard, taken in the Shamshy co‐managed nature protected area, Kyrgyzstan: (A) 2 horses, one feeding on *Myricaria* sp. and the other on *Salix* sp. (25th September 2022); (B) a domestic dog sniffing *Myricaria* sp. (4th June 2023); (C) a Siberian ibex sniffing *Myricaria* sp. (24th October 2024).

The camera documented three separate occurrences of the snow leopard on non‐consecutive days, during which the animal was observed feeding on *Myricaria* sp.: 9th December 2022, at 16:40 h (1 video; Figure 2A‐1,2,3), 24th May 2023, at 19:49 h (4 videos; Figure 2B‐1,2,3), and 25th November 2024, at 16:32 h (10 videos; Figure 2C‐1,2,3). All three occurrences were identified as the same adult male individual based on its coat pattern, an individual named as OMUTA that has been documented in this protected area since 2018. As shown in Figure 2A‐1, the snow leopard exhibited sniffing behavior toward *Salix* sp., but did not ingest it. Feeding on *Myricaria* sp. was observed in 14 out of 15 video recordings, combining three occurrences (Table 1). Prior to biting, the animal sniffed the *Myricaria* sp. branches, a behavior recorded in 11 of the 15 videos (Table 1). As illustrated in Figure 2A‐3,B‐2,C‐2, the snow leopard gnawed and consumed the branch progressively from the distal tip toward the proximal base near the ground. Additionally, head‐rubbing against *Myricaria* sp. was observed following branch biting in 7 of the 15 recordings (Figure 2B‐3,C‐3, Table 1).

Horses were recorded on 6 non‐consecutive days (50 video recordings in total), including instances when they appeared with humans (e.g., being ridden). However, feeding on *Myricaria* sp. was observed only on a single day (25th September 2022, between 14:00 and 15:00 h), during which multiple individuals were recorded feeding (14 videos; Figure 3A). As noted above, the snow leopard consumed *Myricaria* sp. during winter and early spring, when the plant consisted mostly of stubbles and stems, whereas horses fed on it only during the leaf‐bearing period within the study period. On this day, the 18 horses passed in front of the camera; seven were observed feeding on the leaves and twigs of *Myricaria* sp. until the plants were completely stripped of their leaves, leaving only the branches. Notably, five of these seven individuals also consumed *Salix* sp. alongside *Myricaria* sp. (Figure 3A). While the horses primarily grazed on herbaceous vegetation, these woody plants served as supplementary browse.

In several instances where horses appeared with humans, domestic dogs ( *Canis lupus familiaris* ; hereafter “dogs”) were also present. Four individual dogs were recorded in seven video recordings across five non‐consecutive days. In three of these videos—two recorded on 4th June 2023 and one on 26th October 2024—one dog on each day was observed sniffing *Myricaria* sp. (Figure 3B). At these times, the *Myricaria* sp. had no leaves, consisting only of branches, and no feeding behavior on *Myricaria* sp. was observed.

Ibexes, a primary prey species of snow leopard, were also captured by the camera. Across 11 non‐consecutive days, 25 video recordings documented 67 individuals in two solitary sightings (one solitary sub‐adult male and one solitary adult male) and 11 groups (three female‐with‐calf groups, one sub‐adult male group, three adult male groups, and four mixed‐sex groups). Grazing on herbaceous vegetation was observed in three recordings, however no feeding on *Myricaria* sp. was observed. Sniffing behavior directed toward *Myricaria* sp. occurred in 4 videos across 3 non‐consecutive days during the leafless season (6th and 17th October 2022, and 24th October 2024) (Figure 3C).

## 4 Discussion

To our knowledge, this study provides the first camera‐trap evidence of *Myricaria* sp. consumption by snow leopards. The presence of numerous small *Myricaria* sp. branch fragments in fecal samples (Figure 1A‐1,2) is consistent with the video sequences in which the snow leopard actively bit branches and consumed them while interacting with the *Myricaria* sp. Although opportunistic and limited in scope, such observations can broaden inferences about behavioral diversity in elusive large carnivores.

Why would a snow leopard consume woody plant material? In domestic cats, plant eating is generally regarded as an instinctive behavior rather than a response to illness or dietary deficit (Hart et al. 2021). One hypothesis is facilitation of hairball expulsion by boosting dietary fiber intake and gastrointestinal motility (Loureiro et al. 2014; Bensel et al. 2025). Given the dense pelage of snow leopards (Heptner 1992) and personal observation of substantial self‐ingested hair in the feces of captive individuals (Kinoshita et al. 2009; Kinoshita et al. 2011), plant intake could plausibly assists in fur elimination. However, a study of captive snow leopards—some of which voluntarily consumed plants growing in their enclosures or chewed on wooden structures (e.g., benches), though *Myricaria* sp. was not present—reported no significant effect of plant consumption on defecation or vomiting‐induced fur expulsion (Yoshimura et al. 2020). In addition to the hair evacuation hypothesis, several studies have discussed the self‐medication hypothesis (Hart 2008; Hart et al. 2021; Yoshimura et al. 2021). These studies suggest that felids may consume herbaceous plants to expel intestinal parasites from the gastrointestinal tract. Although these specific studies do not explicitly address the consumption of woody plants, the utilization of *Myricaria* sp. could potentially serve a similar functional purpose. Co‐occurrence of intestinal parasites with plants has been observed in various mammals, including primates such as chimpanzees (Huffman and Caton 2001) and numerous species within the order Carnivora (Franck and Farid 2020). Hart and Hart (2018) suggest that the consumption of fibrous, non‐digestible plant material helps to physically expel intestinal parasites by increasing gut motility. Some *Myricaria* species possess bioactive properties (Kletter et al. 2008; Liu et al. 2015, 2016, 2024, 2025; Sharma et al. 2024), suggesting potential medicinal benefits; this, however, requires targeted chemical and physiological tests.

A notable difference in consumption style was observed between horses and the snow leopard. While horses selectively consumed the leaves and thin twigs of *Myricaria* sp. and *Salix* sp. during the leaf‐bearing period, the snow leopard consumed *Myricaria* sp. by gnawing and consuming the branch down to its base, even when the plant consisted mostly of woody stubbles. This suggests that while horses utilize these woody plants as supplementary leafy forage, the snow leopard's consumption involves a more intensive ingestion of structural plant parts. Wood‐chewing is a recognized behavior in domestic cats and horses, and provision of hay or other fibrous feeds can reduce wood‐chewing (Houpt 1981; Demontigny‐Bedard et al. 2016). Metabarcoding of wild snow leopard scats indicated that samples containing *Myricaria* sp. often lacked prey DNA, raising the possibility that plant intake occurs when the digestive tract is relatively empty (Yoshimura et al. 2024). While *Salix* sp. was also present in the study area, the reported DNA metabarcoding results showed that *Myricaria* sp. was the predominantly detected woody plant in snow leopard feces, with *Salix* sp. appearing only minimally. Consequently, our discussion focuses on the potential drivers of *Myricaria* sp. consumption as a primary woody supplement. Plant consumption may also reflect a compensatory response to reduced opportunities for chewing during prey scarcity. Chewing per se may also provide oral motor stimulation or stress relief; in rodents, gnawing wooden sticks mitigates stress related gastric and behavioral outcomes (Koizumi et al. 2011), and in dogs chewing contributes to oral hygiene (Hennet 2001). Such functions could be evolutionarily significant, supporting both stress regulation and dental health. Furthermore, the snow leopard is known for its high dietary plasticity, adapting its prey selection to local and seasonal availability (Koju et al. 2023). While traditional diet studies based on morphological fecal analysis can be subject to species misidentification biases (Weiskopf et al. 2016), our direct video evidence combined with DNA metabarcoding provides an unambiguous record of botanical resource use. Importantly, in our sequences the snow leopard did more than gnaw: it severed multiple branches to their base and swallowed the fragments. Fecal evidence corroborates true ingestion rather than incidental swallowing. As shown in Figure 1A‐1,2, macroscopic woody fragments of *Myricaria* sp. were directly observed within the snow leopard scat matrix. This physical evidence, along with the DNA metabarcoding results (Yoshimura et al. 2024), confirms that the snow leopard intentionally severs and swallows the branches, distinguishing this behavior from incidental ingestion. Ingestion of woody material could provide transient satiety or other benefits, but the underlying driver remains unresolved.

We additionally recorded the snow leopard rubbing its head against the *Myricaria* sp. following feeding. Head‐rubbing is widely interpreted as a form of scent marking via facial glands (Soini et al. 2012) and is often observed alongside urine spraying in snow leopards (Freeman 1983; Kinoshita et al. 2009; Macri and Patterson‐Kane 2011; Krofel et al. 2025). Our observations suggest that *Myricaria* sp. may also function as a marking substrate. Analogously, specific plants such as catnip ( *Nepeta cataria* ) elicit rubbing/rolling in domestic cats (Todd 1963; Bol et al. 2022), potentially via plant volatiles that mimic feline pheromones (Bol et al. 2022) or confer mosquito‐repellent benefits (Uenoyama et al. 2021)—though the latter seems less likely to apply in high‐altitude snow leopard habitats. Notably, sniffing directed at *Myricaria* sp. was also observed in horses, dogs, and ibex, whereas ingestion was confined to snow leopards and horses, indicating that *Myricaria* sp. may mediate roles beyond forage, possibly linked to olfactory communication or other behavioral functions.

Our study represents the first direct observation of a wild snow leopard consuming and subsequently rubbing against a specific plant species. Although these findings are based on a single individual, this specific male is a “long‐term resident” that adds value to the observation as a potentially established behavioral trait rather than a transient one. Additionally, while the lack of local vegetation data precludes a formal assessment of dietary preference, the observations still provide a pioneering basis for future long‐term and multi‐site research to confirm the prevalence of such behaviors across the species' range. Dedicated studies are required to understand plant‐related behaviors in felids and their association with stress relief, pheromonal communication, or other adaptive purposes. Future research could incorporate chemical and physiological analyzes to clarify the function of these interactions. Also given that even within the Felidae family, the purposes of plant for consumption and communication may vary between species, it is important to identify the specific plant species used by wild individuals of each species. Further studies may investigate compounds that the felids are targeting and if these behaviors reflect a broader ecological or physiological strategy among carnivores.

## Author Contributions

**Kodzue Kinoshita:** conceptualization (equal), data curation (equal), funding acquisition (equal), writing – original draft (equal). **Hiroto Yoshimura:** data curation (equal), writing – review and editing (equal). **Kubanychbek Zhumabai uulu:** data curation (equal), project administration (equal), writing – review and editing (equal). **Koustubh Sharma:** project administration (equal), writing – review and editing (equal). **Aibek Sergek uulu:** data curation (equal). **Emil Djaparov:** data curation (equal). **Kurmanbek Tolok uulu:** data curation (equal). **Dale M. Kikuchi:** conceptualization (equal), data curation (equal), writing – review and editing (equal).

## Funding

This work was supported by Japan Prize Foundation, JSPS Bilateral Program (JPJSBP120249923), and JSPS KAKENHI Grant‐in‐Aid for Scientific Research (B) (20H03008 and 24K03130).

## Conflicts of Interest

The authors declare no conflicts of interest.

## Supporting information


**Table S1:** Standardized ethogram.

## Data Availability

The supplementary data and videos supporting the findings of this study are available from the Dryad Digital Repository at https://doi.org/10.5061/dryad.905qftv0r and Zenodo (https://doi.org/10.5281/zenodo.19751807), respectively.
